# Biaxial strain tuned upconversion photoluminescence of monolayer WS_2_

**DOI:** 10.1038/s41598-024-54185-8

**Published:** 2024-02-15

**Authors:** Shrawan Roy, Xiaodong Yang, Jie Gao

**Affiliations:** 1 Department of Mechanical and Aerospace Engineering, Missouri University of Science and Technology, Rolla, MO 65409 USA; 2 Department of Mechanical Engineering, Stony Brook University, Stony Brook, NY 11794 USA

**Keywords:** Upconversion photoluminescence, Biaxial strain, Monolayer WS_2_, Integrated optics, Two-dimensional materials

## Abstract

Monolayer tungsten disulfide (1L-WS_2_) is a direct bandgap atomic-layered semiconductor material with strain tunable optical and optoelectronic properties among the monolayer transition metal dichalcogenides (1L-TMDs). Here, we demonstrate biaxial strain tuned upconversion photoluminescence (UPL) from exfoliated 1L-WS_2_ flakes transferred on a flexible polycarbonate cruciform substrate. When the biaxial strain applied to 1L-WS_2_ increases from 0 to 0.51%, it is observed that the UPL peak position is redshifted by up to 60 nm/% strain, while the UPL intensity exhibits exponential growth with the upconversion energy difference varying from − 303 to − 120 meV. The measured power dependence of UPL from 1L-WS_2_ under biaxial strain reveals the one photon involved multiphonon-mediated upconversion mechanism. The demonstrated results provide new opportunities in advancing TMD-based optical upconversion devices for future flexible photonics and optoelectronics.

## Introduction

Monolayer tungsten disulfide (1L-WS_2_) is a direct bandgap atomic-layered semiconductor material^1–3^, belonging to the transition metal dichalcogenides (TMDs) family. 1L-WS_2_ has been widely studied due to its promising physical properties such as high quantum yield^4,5^, sizable spin–orbit coupling^6^, and wafer-scale uniform growth^7,8^. Rather than the photoluminescence (PL) observed in 1L-WS_2_ with the above-bandgap excitation, upconversion photoluminescence (UPL) can also be achieved in 1L-WS_2_ with the below-bandgap excitation at room temperature^9,10^. UPL represents an anti-Stokes process with the emitted photons having higher energy compared to the absorbed photons, and the phenomenon has been studied with different types of materials such as quantum wells^11^, quantum dots^12^, rare-earth-doped materials^13^, organic dyes^14,15^, and 1L-TMDs^9,16–18^. UPL emission has wide applications in many fields for advancing future optoelectronics such as lasers^19^, displays^20^, photovoltaics^21^, bioimaging^22,23^, and optical refrigeration^24^.

Tunable optical and optoelectronic properties of 1L-TMDs can be achieved by using various tuning methods such as electrostatic doping^25^, chemical treatment^2,3,26,27^, heterostructure forming^10,28,29^, and strain engineering^30–32^. Among these methods, strain engineering is one effective approach to adjust the crystal lattice and band structure of 1L-TMDs, so that their optical responses are modulated such as PL emission, Raman scattering, and optical absorption^30–36^. Strain engineering has been carried out on TMDs by multiple ways such as bending TMD layers on flexible substrates^30,31,34–37^, forming ripples and wrinkles^32,33,38^, and transferring TMD layers on patterned substrates^39,40^. The study of strain engineered UPL emission from 1L-TMDs is highly demanded for realizing future tunable optical upconversion devices and flexible optoelectronic platforms. However, the strain tuned UPL emission in 1L-TMDs particularly in 1L-WS_2_ has not been comprehensively investigated until now.

Here, biaxial strain tuning of UPL emission from exfoliated 1L-WS_2_ at room temperature is demonstrated. By utilizing the bending and indentation method, biaxial strain is applied to the exfoliated 1L-WS_2_ flakes transferred on flexible polycarbonate (PC) cruciform substrate. When the biaxial strain applied to 1L-WS_2_ increases from 0 to 0.51%, the UPL peak positions are redshifted with 54 nm/% strain and 60 nm/% strain at the excitations of 685 nm and 725 nm, respectively, which are equivalent to the strain tuning gauge factors of 163 meV/% strain and 179 meV/% strain. Meanwhile, biaxial strain tuned UPL intensity in 1L-WS_2_ is demonstrated to follow an exponential growth function with the upconversion energy difference in a wide range from − 303 to − 120 meV, with the enhancement of UPL intensity in nearly 75 times. The power dependence of UPL from 1L-WS_2_ under biaxial strain at the excitation wavelengths of 685 nm and 725 nm elucidates the one photon involved upconversion process assisted by multiphonon absorption. These results will offer new possibilities in facilitating TMD-based optical upconversion devices for future applications in infrared sensing, night vision, photodetection, flexible photonics and optoelectronics.

## Results and discussion

**Figure 1:**
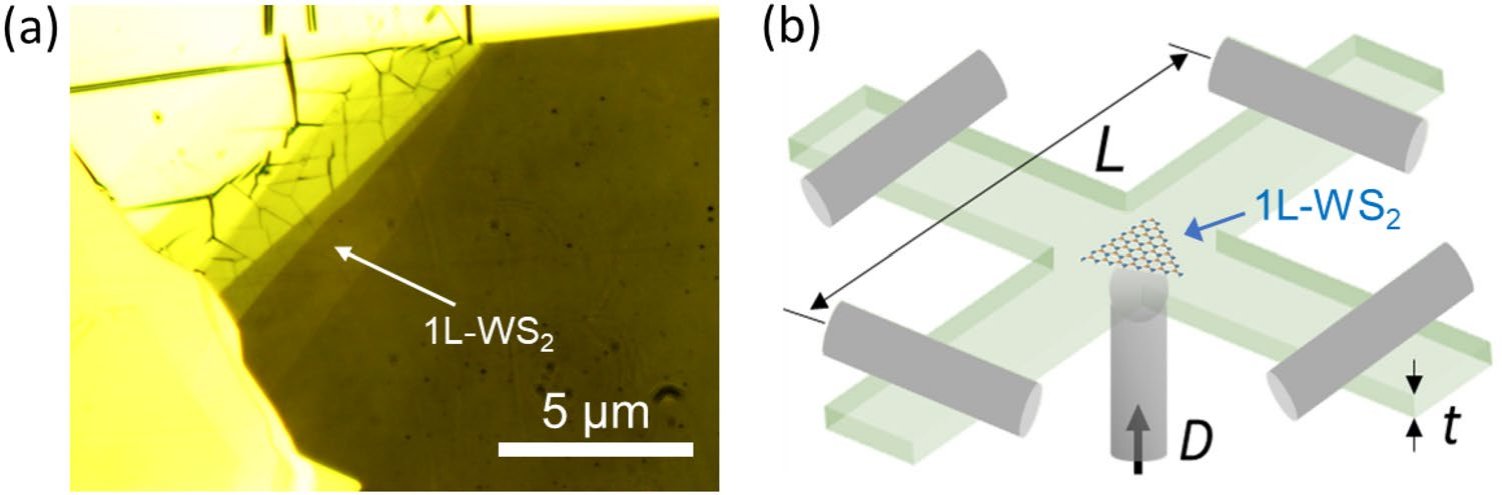
(**a**) Optical image of an exfoliated 1L-WS_2_ flake transferred on flexible PC cruciform substrate. (**b**) Schematic diagram for the biaxial strain apparatus with the bending and indentation method.

The optical reflection microscope image of the mechanically exfoliated 1L-WS_2_ flake transferred on flexible PC cruciform substrate is displayed in Fig. 1a. Figure 1b shows the schematic diagram of the biaxial strain apparatus with the bending and indentation method^37,41^, where the biaxial strain is applied to 1L-WS_2_ flake transferred at the center area of PC cruciform substrate pinned at its four edges by metal rods. The PC cruciform substrate is inserted between the rod assembly and the central sphere indenter. As the central sphere indenter deflects the substrate upwards, the equi-biaxial tensile strain is applied on the top surface of the substrate. The biaxial strain applied to the 1L-WS_2_ flake is described by *ε* = *3Dt/L*^*2*^, where *L* is the distance between the two opposite pin edges (*L* = 25.4 mm), *t* is the thickness of the PC cruciform substrate (*t* = 0.25 mm), and *D* is the displacement of the central sphere indenter.

**Figure 2:**
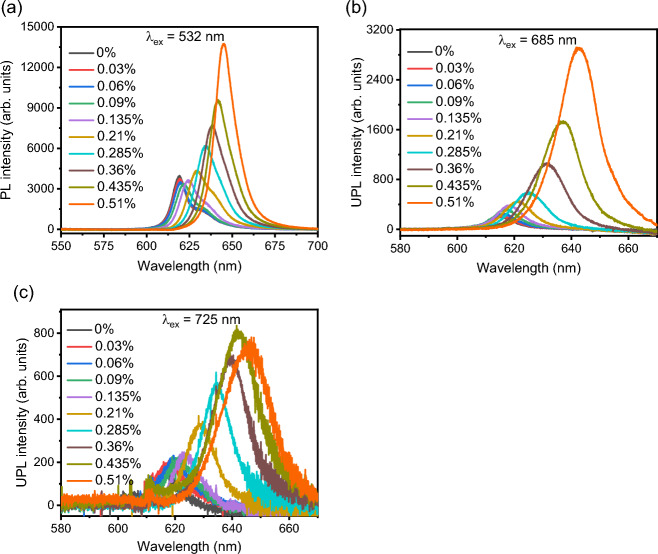
(**a**) Biaxial strain tuned PL spectra of 1L-WS_2_ excited at 532 nm. (**b**, **c**) Biaxial strain tuned UPL spectra excited at 685 nm and 725 nm, respectively.

The biaxial strain tuned PL spectra excited at 532 nm and UPL spectra excited at 685 nm and 725 nm for the 1L-WS_2_ sample at room temperature are presented in Fig. 2. In Fig. 2a, the PL peak position is observed at around 619 nm without strain, which matches with the typical PL spectrum of 1L-WS_2_^1–5^. The PL peak position continuously gets redshifted as the biaxial strain increases from 0 to 0.51%, with approximately 4 times enhancement in the PL intensity. In Fig. 2b,c, the UPL peak positions without strain under the excitation wavelengths of 685 nm and 725 nm are around 616 nm, which is consistent with the PL peak position. It is observed that the UPL peak positions are also redshifted under the influence of increasing levels of applied biaxial strain, while the enhancement of UPL intensity is nearly 15 and 5 times under the excitation wavelengths of 685 nm and 725 nm, respectively. It is noted that the UPL intensity at 0.51% strain under 725 nm excitation is slightly lower compared to that at 0.435% strain, which may be resulted from the change in sample condition after several cycles of applied strain.

**Figure 3:**
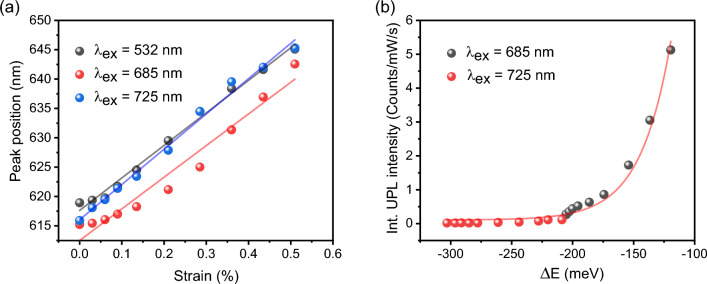
(**a**) PL and UPL peak positions as a function of the biaxial strain applied to 1L-WS_2_ under different excitation wavelengths. (**b**) Dependence of the integrated UPL intensity on the strain tuned upconversion energy difference under the excitations of 685 nm and 725 nm.

Figure 3a summarizes the PL and UPL peak positions as a function of the biaxial strain applied to 1L-WS_2_ under different excitation wavelengths, showing a linear dependence of the peak position shift on the strain level. The PL peak position redshift of 55 nm/% strain is obtained at the excitation of 532 nm with the strain tuning gauge factor of 163 meV/% strain, which is similar to the results reported in the previous work^37,42^. The UPL peak position redshifts at the excitations of 685 nm and 725 nm are 54 nm/% strain and 60 nm/% strain, corresponding to the strain tuning gauge factors of 163 meV/% strain and 179 meV/% strain, respectively. The slight difference between the strain tuning gauge factors at different excitation wavelengths is due to different strain transfer conditions between 1L-WS_2_ and substrate during each cycle of applied strain^30^. For the biaxial strain varied from 0 to 0.51%, the upconversion energy difference between the excitation photon energy and the UPL emission energy *ΔE* = *ℏω*_*ex*._ − *ℏω*_*UPL*_ is tuned from − 205 to − 120 meV at the excitation of 685 nm, and from − 303 to − 209 meV at the excitation of 725 nm. Figure 3b shows the dependence of the integrated UPL intensity from 1L-WS_2_ on the strain tuned upconversion energy difference in the wide range of − 303 to − 120 meV under the excitations of 685 nm and 725 nm at room temperature. It shows that the UPL intensity follows an exponential growth function with the upconversion energy difference, with the enhancement of UPL intensity in nearly 75 times. The UPL intensity can be described by the Boltzmann function as ${I}_{UPL}\propto \mathit{exp}\left(-\left|\Delta E\right|/{k}_{B}T\right)$, where $\left|\Delta E\right|$ is the upconversion energy gain, ${k}_{B}$ is the Boltzmann constant, and *T* is the room temperature at 298 K. The effective phonon number involved in the multiphonon-assisted upconversion process in 1L-WS_2_ is approximately estimated from 6 to 2, based on the ratio between |*ΔE*| from 303 to 120 meV and the phonon energy of 52 meV for the A_1g_ transverse optical phonon in 1L-WS_2_.

**Figure 4:**
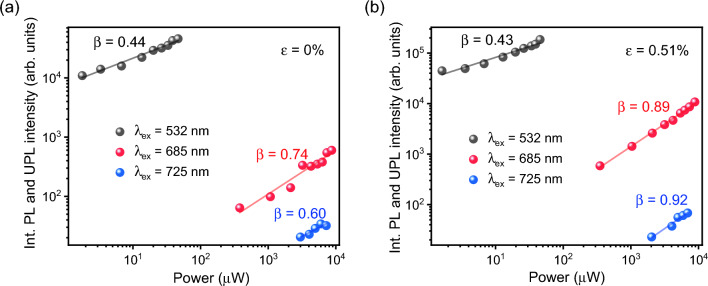
Power dependent integrated PL and UPL intensities in a log–log scale under the excitations of 532 nm, 685 nm, and 725 nm at the biaxial strain of (**a**) 0% and (**b**) 0.51%.

The power dependent PL and UPL intensities from 1L-WS_2_ excited at 532 nm, 685 nm, and 725 nm are plotted in a log–log scale in Fig. 4a,b at the biaxial strain of 0% and 0.51%, respectively. The power law of *I* = *αP*^*β*^ is used to fit the PL and UPL intensities, where *P* is the excitation power, *α* is the fitting parameter, and *β* is the exponent. It is observed that the *β* values for PL emission at both strain levels show a sublinear power dependence, which is due to the presence of nonradiative exciton–exciton annihilation at high excitation intensity. The *β* values for UPL emission at 0% strain also exhibit a sublinear power dependence at the excitations of 685 nm and 725 nm. At 0.51% strain, the *β* values increase to around 0.9, which is close to linear power dependence. The sublinear power dependence for the UPL emission may be related to the change of densities of phonons and exciton complexes^9^. The power dependence of UPL emission from 1L-WS_2_ at both strain levels further indicates the one photon involved upconversion photon emission process assisted by multiphonon absorption, rather than any nonlinear optical generation of the upconversion like exciton Auger scattering or two-photon excitation-induced emission^9,16^.

**Figure 5:**
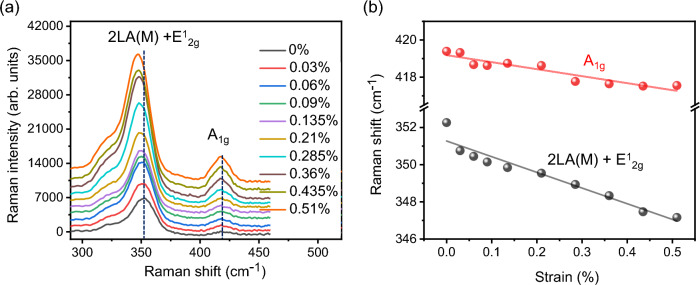
(**a**) Biaxial strain dependent Raman spectra of 1L-WS_2_ excited at 532 nm. The dashed lines mark the Raman peak positions at 0% strain. (**b**) Raman peak positions of A_1g_ and 2LA(M) + E^1^_2g_ modes depending on the biaxial strain.

The biaxial strain dependent Raman spectra of 1L-WS_2_ excited at 532 nm are presented in Fig. 5a. The Raman spectrum of 1L-WS_2_ at 0% strain (black curve) clearly shows two characteristic peaks that correspond to the in-plane longitudinal optical phonon mode (E^1^_2g_) and the second-order acoustic mode [2LA(M)] at the Raman shift of ~ 352 cm^−1^, and the out-of-plane transverse optical phonon mode (A_1g_) at 419 cm^−1^, which are consistent with the previously reported Raman modes of 1L-WS_2_^1–5,43^. It is noteworthy that the observed multiphonon-assisted UPL process is mediated by the out-of-plane A_1g_ transverse optical phonon mode, rather than the in-plane E^1^_2g_ longitudinal optical phonon mode^9,16^. When the biaxial strain gradually increases from 0 to 0.51%, the Raman peak positions of A_1g_ and 2LA(M) + E^1^_2g_ vibrational modes continuously get redshifted by approximately 1.8 cm^−1^ and 5 cm^−1^ respectively, which is resulted from the strain induced crystal symmetry breaking and vibration softening as well as the high strain sensitivity of E^1^_2g_ mode^31,44–46^. Figure 5b plots the linear dependence of the Raman peak positions of A_1g_ and 2LA(M) + E^1^_2g_ modes on the biaxial strain, showing the redshifts of 3.5 cm^−1^/% strain and 9.99 cm^−1^/% strain, respectively. The Raman peak position of A_1g_ mode is nearly three times less sensitive to the applied strain compared to 2LA(M) + E^1^_2g_ mode, giving only slight change in the optical phonon energy around 52 meV during the strain tuned UPL emission from 1L-WS_2_. It is noted that instead of its low strain sensitivity, the A_1g_ mode of 1L-WS_2_ is highly sensitive to the electronic changes like electrostatic doping^45,46^. The overall Raman intensity is increased about 3.5 times at 0.51% strain compared to that at 0% strain.

## Conclusion

We have demonstrated the biaxial strain tuning of UPL emission from mechanically exfoliated 1L-WS_2_ on flexible PC cruciform substrate by utilizing the bending and indentation method. The UPL peak positions get redshifted by 54 nm/% strain and 60 nm/% strain under the excitation wavelengths of 685 nm and 725 nm as the biaxial strain varies from 0% to 0.51%, which provide the strain tuning gauge factors of 163 meV/% strain and 179 meV/% strain, respectively. The strain tuned UPL intensity follows an exponential function with the upconversion energy difference in a range of − 303 to − 120 meV, with the UPL intensity enhancement of nearly 75 times. The power dependence of UPL emission from 1L-WS_2_ under biaxial strain indicates the one photon involved upconversion process mediated by multiphonon absorption. The demonstrated results will enable new opportunities in promoting TMD-based tunable photon upconversion devices used for many applications such as infrared sensing, imaging, night vision, and flexible optoelectronics.

## Methods

### Sample preparation

1L-WS_2_ flakes were mechanically exfoliated from a bulk WS_2_ crystal (2D Semiconductors) with scotch tape method. Bulk WS_2_ crystal was first deposited on scotch tape and exfoliated many times to obtain thin layers including monolayers, then the tape was gently placed on a small piece of polydimethylsiloxane (PDMS) film attached on glass slide for approximately 45 min. Next, the tape was gently removed to obtain multilayer and 1L-WS_2_ flakes on PDMS film. 1L-WS_2_ flakes were confirmed by optical microscopic image, PL, and Raman spectra. The cruciform substrate has a length of 60 mm and the width of 8 mm, which was cut from a 0.25 mm thick PC board. Finally, the exfoliated 1L-WSe_2_ flakes on PDMS film were transferred to the center area of PC cruciform substrate with the dry transfer method based on an optical microscope equipped with a micromanipulator^47^.

### Optical measurements

PL and Raman spectra of 1L-WS_2_ flakes on PC cruciform substrate were characterized with a 532 nm excitation laser by recording the back reflected signal from a 50 × objective lens (NA = 0.42) that was coupled into a spectrometer (Horiba, iHR 550) through a beam splitter and a 532 nm ultrasteep longpass edge filter. The UPL spectra were acquired by using the same measurement setup with continuous-wave excitation lasers at the wavelengths of 685 nm and 725 nm, and the corresponding 675 nm and 700 nm shortpass filters.

## Data Availability

The datasets generated during and/or analyzed during the current study are available from the corresponding author on reasonable request.
